# Drug-Polymers Composite Matrix Tablets: Effect of Hydroxypropyl Methylcellulose (HPMC) K-Series on Porosity, Compatibility, and Release Behavior of the Tablet Containing a BCS Class I Drug

**DOI:** 10.3390/polym14163406

**Published:** 2022-08-19

**Authors:** Namon Hirun, Pakorn Kraisit

**Affiliations:** Thammasat University Research Unit in Smart Materials and Innovative Technology for Pharmaceutical Applications (SMIT-Pharm), Faculty of Pharmacy, Thammasat University, Pathumthani 12120, Thailand

**Keywords:** matrix tablets, hydroxypropyl methylcellulose (HPMC), propranolol hydrochloride, porosity, Korsmeyer–Peppas model, Higuchi model

## Abstract

The purpose of this research was to see how the physicochemical properties and porosity of matrix tablets containing various types of hydroxypropyl methylcellulose (HPMC) K series affected the release of propranolol hydrochloride (PNL). PNL is a class I drug (high solubility and permeability) according to the Biopharmaceutics Classification System (BCS), making it an excellent model drug used for studying extended-release drug products. The direct compression method was used to prepare the HPMC-based matrix tablets. PNL and the excipients were found to be compatible using Fourier transform infrared spectroscopy (FTIR), powder X-ray diffraction (PXRD), and differential scanning calorimetry (DSC). The surfaces of all the compressed HPMC-based matrix tablets were rough, with accumulated particles and small holes. The compressed HPMC-based matrix tablet porosity was also determined by using mercury porosimetry. The compressed HPMC-based matrix tablets made of low viscosity HPMC had tiny pores (diameter < 0.01 μm). The shorter polymeric chains are more prone to deformation, resulting in a small pore proportion. The compressed HPMC-based matrix tablets sustained the release of PNL for over 12 h. The release exponent values (n), which reflect the release mechanism of the drug from the tablets, ranged from 0.476 to 0.497. These values indicated that the release was governed by anomalous transport. The compressed HPMC-based matrix tablets have the potential for a sustained release of PNL.

## 1. Introduction

**Scheme 1:**
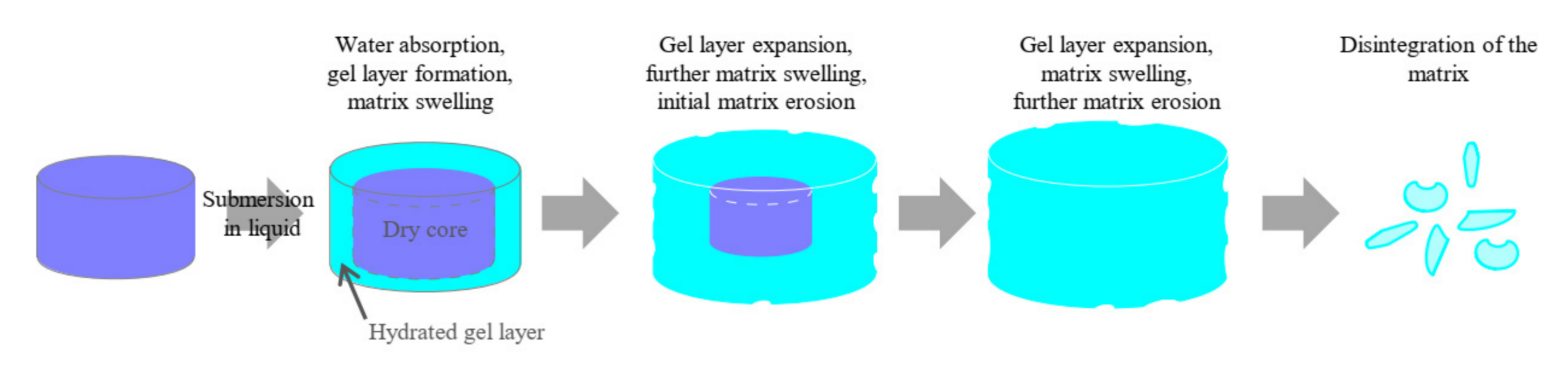
Possible evolution of a hydrophilic matrix tablet over time after submersion in liquid.

Hydrophilic matrices are monolithic systems of a matrix containing one or more hydrophilic excipients and an active pharmaceutical ingredient [1]. From a practical aspect, a direct compression hydrophilic matrix tablet is one of the simplest ways of developing an oral extended-release dosage form. The direct compression hydrophilic matrix tablet is typically favored for manufacturing because of its numerous advantages, such as current technology, ease of manufacture, and low production cost [2,3,4]. Hydrophilic polymers are the most used hydrophilic excipients in developing compressed hydrophilic matrices. Following exposure to water, the hydrophilic polymers swell and form a gel layer [5,6]. The gel layer regulates the drug release mechanism by controlling water entry and drug molecules’ diffusion [7]. In some hydrophilic matrices, an anomalous transport could occur [8]. This phenomenon corresponds to the combined effect of diffusion with polymer relaxation and/or matrix erosion [9]. In combination with the expansion of the gel layer, other phenomena and characteristics such as matrix erosion, the internal structure of the matrix, and matrix porosity determine the release behavior of the drug from the hydrophilic matrix [1,4,10]. The interplay of hydration, gelling, swelling, erosion, and maybe even the final breakup of the dosage form (Scheme 1) can cause drug release from a hydrophilic matrix tablet [11]. The hydrophilic matrix absorbs water and forms the hydrated gel layer around the dry core after being immersed in the fluid. Furthermore, the hydrated tablet may swell. The drug’s release may be affected over time by the growth of the hydrated gel layer and the migration of fluid into the tablet, resulting in the loss of the dry core. Some hydrophilic matrices may also erode and disintegrate over time, potentially resulting in drug release. To develop an oral extended-release matrix, understanding the impact of the hydrophilic polymer in releasing drugs from the hydrophilic matrix is crucial.

Hydroxypropyl methylcellulose (HPMC), also called hypromellose, has a cellulosic backbone that is partly substituted with methoxy and hydroxypropoxy groups. It is an enzyme-resistant, water-soluble hydrophilic polymer that is stable over a pH range of 3.0–11.0 [12]. HPMC is an inactive pharmaceutical ingredient for use in oral, ophthalmic, nasal, and topical formulations, and it is approved by the US Food and Drug Administration [12,13,14]. There are several commercially available HPMC grades; the substituent’s viscosity and extent differ among the various HPMC grades. In the development of drug delivery technology, the study of hydrophilic polymers has received considerable attention. The hydrophilic matrix’s performance depends on the polymer type and viscosity grade, the presence of certain excipients, and even on factors associated with the material manufacturing, such as porosity [1,2,15]. Numerous researchers have studied the effects of different viscosity grades and degrees of substitution of HPMC on the compaction properties and drug release behavior of matrices [15,16,17,18]. Moreover, the effect of the type of preparation procedure used to make a drug-loaded matrix incorporating HPMC and another excipient, lactose, on drug release was recently studied [19]. Compared to the physical mixture, it has been shown that co-processed excipients did not affect the drug release rate of tablets manufactured by direct compression [19]. The impact of HPMC viscosity grades on drug release has also been investigated for buccal delivery systems [20]. However, none of them have looked at the impact of HPMC viscosity grades on drug release and the porosity of monolithic matrices made under constant compression force for oral drug administration. The information about the effects of HPMC grades on the porosity and release characteristics of the hydrophilic matrix can be beneficial in interpreting the behavior of the HPMC-based system when utilized to manufacture oral drug delivery tablets.

This study used different HPMC viscosity grades to prepare the compressed HPMC-based matrix tablets at the constant compaction force, and the manufactured tablets were characterized. Propranolol HCl (PNL), a beta-blocker, was used as a model drug. PNL is a class I drug (high solubility and permeability) according to the Biopharmaceutics Classification System (BCS), making it an excellent model drug used for the study of extended-release drug products [21,22]. The compatibility among the drug, HPMC, and other excipients was verified using Fourier transform infrared spectroscopy (FT-IR), powder X-ray diffraction (PXRD), and differential scanning calorimetry (DSC). The morphology and porosity of the compressed HPMC-based matrix tablets were examined using scanning electron microscopy (SEM) and mercury porosimetry. Finally, the release of the PNL from the compressed HPMC-based matrix tablets was determined.

## 2. Materials and Methods

### 2.1. Materials

**Figure 1:**
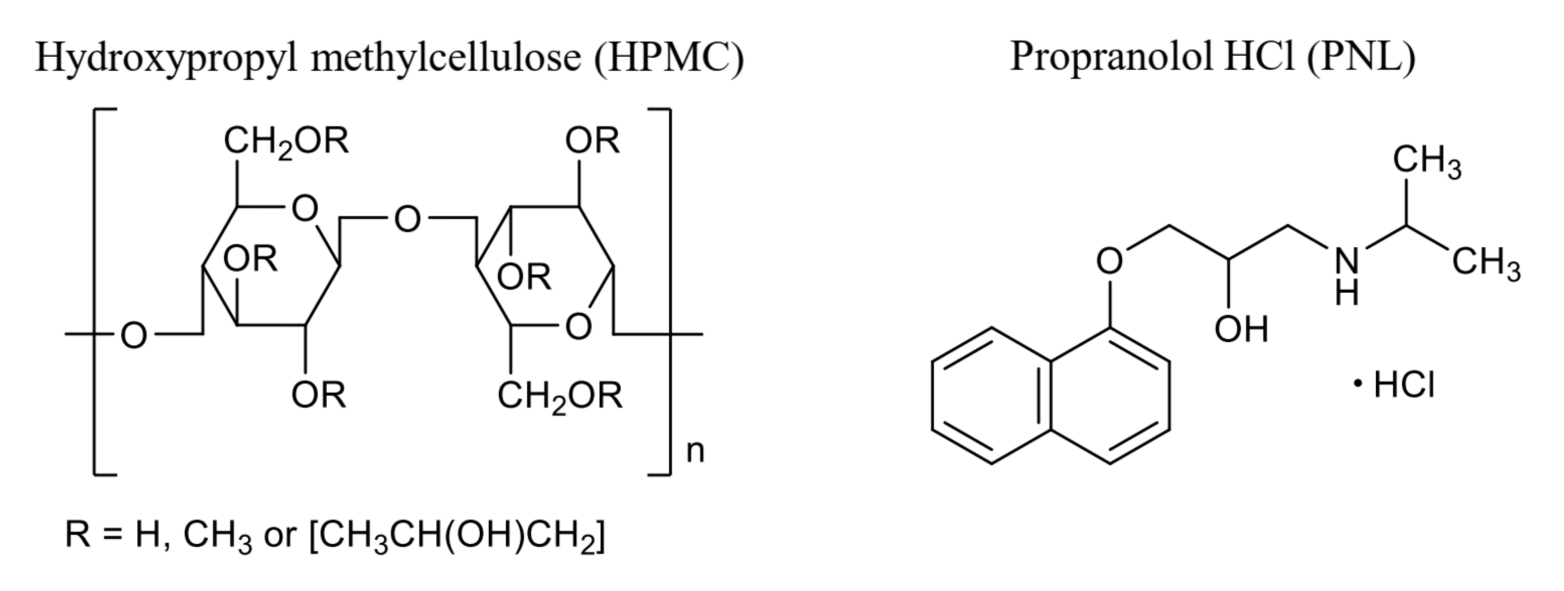
The chemical structures of HPMC and PNL.

Various grades of HPMC (Methocel^®^) such as K4M (H-K4M), K15M (H-K15M), and K100M (H-K100M) were manufactured by Dow Chemical Company (Midland, MI, USA) and kindly supported by Rama Production Co., Ltd. (Bangkok, Thailand). The viscosity of K4M, K15M, and K100M is 4000, 15,000, and 100,000 mPa·s, respectively [23]. PNL was purchased from PC Drug Co., Ltd. (Bangkok, Thailand). The chemical structures of HPMC and PNL are shown in Figure 1. All other chemicals were of analytical grade and were used in their original state.

### 2.2. Preparation of Compressed HPMC-Based Matrix Tablets

Preparing compressed HPMC-based matrix tablets via direct compression was adapted from a previous study in Kraisit P. [24]. Pharmaceutical excipients such as microcrystalline cellulose and dibasic calcium phosphate dihydrate are generally used as diluents in tablet formulation. Combining them can help improve hardness and compressibility [24,25]. The tablets, with a total weight of 400 mg, contained 35% dibasic calcium phosphate dihydrate, 20% PNL (80 mg/tablet), 19% microcrystalline cellulose, 8% sodium starch glycolate, 2% talcum, 1% magnesium stearate, and 15% HPMC. Various concentrations and types of HPMC (Table 1) were combined with precise amounts of microcrystalline cellulose, dibasic calcium phosphate dihydrate, and PNL. After the compounds were uniform, they were manually mixed for 5 min in a polybag with the remaining ingredients. A hydraulic press was used to compress the mixture with a 9.5-mm diameter flat-faced punch at a force applied of 1 ton and a holding time of 10 s (Model 15011, Specac, Fort Washington, PA, USA). Prior to the examination, the tablets were kept in a vacuum desiccator at room temperature (*ca.* 28 °C), and humidity was around 35 %.

### 2.3. Scanning Electron Microscopy (SEM)

The top view of the compressed HPMC-based matrix tablets and the broken-tablets side view were analyzed morphologically using SEM (model JSM-5410LV, Jeol, Tokyo, Japan) at a 20 kV acceleration voltage. Prior to obtaining the micrographs, the tablets were adhered to a metal stub with double-sided adhesive tape and covered with a fine gold layer under a vacuum.

### 2.4. Fourier Transform Infrared Spectroscopy (FTIR)

The FTIR spectra of samples were determined using FTIR spectrophotometer (Nicolet, Magna 750, Waltham, MA, USA). The sample was ground, blended with KBr powder, and compressed at a pressure of 5 tons using a hydraulic press. The disc was placed in a sample holder and scanned from 4000 to 400 cm^−1^ at a resolution of 4 cm^−1^.

### 2.5. Powder X-ray Diffractometry (PXRD)

The samples’ X-ray diffraction patterns were determined at 30 kV and 15 mA using a MiniFlex II (Rigaku, Tokyo, Japan). The relative intensities were determined over a 5°–45° 2θ range using a Cu Kα radiation wavelength of 1.5406 Å.

### 2.6. Differential Scanning Calorimetry (DSC)

The thermal characteristics of the samples were determined using the DSC 8000 (PerkinElmer, Waltham, MA, USA). Two to five mg of sample were accurately weighed and sealed in a solid aluminum pan. The measurement temperature ranged from 30 to 300 °C, with a heating rate of 10 °C/min. The nitrogen purge rate was set to 20 mL/min for the analysis.

### 2.7. Porosity

The total pore surface area, mean pore diameter, and pore diameter were determined using a Mercury intrusion porosimeter (AutoPore V, Micromeritics, Norcross, GA, USA) operating at pressures ranging from 0.5 to 60,000 psi. Each compressed HPMC-based matrix tablet was removed from a vacuum desiccator and precisely weighed with an analytical balance. The tablet was placed into a penetrometer and filled with mercury. 

Total pore surface area (A_tot_) was calculated by Equation (1) as follows [26]:

(1) $$A_{\mathrm{tot}}=\frac{1}{\gamma \cos\theta \;}\int_{0}^{V_{\mathrm{tot}}}P\cdot \mathrm{dV}$$

where P is the pressure (psia), V is the intrusion volume of mercury (mL g^−1^), V_tot_ is the total intrusion volume of mercury (mL g^−1^), γ the surface tension of mercury (485 dynes cm^−1^), and θ the contact angle of mercury (130°). 

Mean pore diameter (D_m_) was calculated by Equation (2) as follows [26]:

(2) $$D_{m}=4\cdot \;\frac{V_{\mathrm{tot}}}{A_{\mathrm{tot}}}$$

Pore diameter (D) was calculated by Equation (3) as follows [26]:

(3) $$D=\frac{-4\gamma \cos\theta }{P}$$

The 50 percent value derived from cumulative pore size distribution data were used to calculate the median pore diameter [27].

### 2.8. In Vitro Release of Compressed HPMC-Based Matrix Tablets

The basket method at 50 rpm was used to examine the release of PNL from the compressed HPMC-based matrix tablets. The dissolution medium in each vessel used in the release testing was 900 mL of 0.05 M, pH 4.5 of phosphate buffer solution, and kept at 37 °C. The release medium (5 mL) was removed and replaced with an equal amount of fresh dissolution medium at the specified times (1, 3, 6, and 12 h). PNL was analyzed by spectrophotometry at 320 nm (Agilent, model 1100 series, Santa Clara, CA, USA), and PNL was calculated as a percentage of total PNL released (*n* = 6).

### 2.9. Release Data Analysis

The compressed HPMC-based matrix tablets‘ drug release kinetics were compared to Higuchi and Korsmeyer–Peppas equations (Equations (4) and (5), respectively).

M_t_/M_α_ = k_H_t^1^^/2^ (4)

M_t_/M_α_ = kt^n^ (5)

where M_t_/M_α_ denotes the fractional amount of drug released over time (t), k_H_ denotes the Higuchi constant k denotes the Korsmeyer–Peppas constant, and n denotes the drug release mechanism’s release exponent. The drug release mechanism was predicted using the value of n, and the equations with the highest R^2^ were chosen as the best mathematical release model.

### 2.10. Data Analysis

All determinations were performed in at least triplicate. The data are presented as mean values ± SD. The deviation between groups was determined using one-way analysis of variance (ANOVA), with *p*-values less than 0.05 considered significant.

## 3. Results

### 3.1. Compatibility Studies

**Figure 2:**
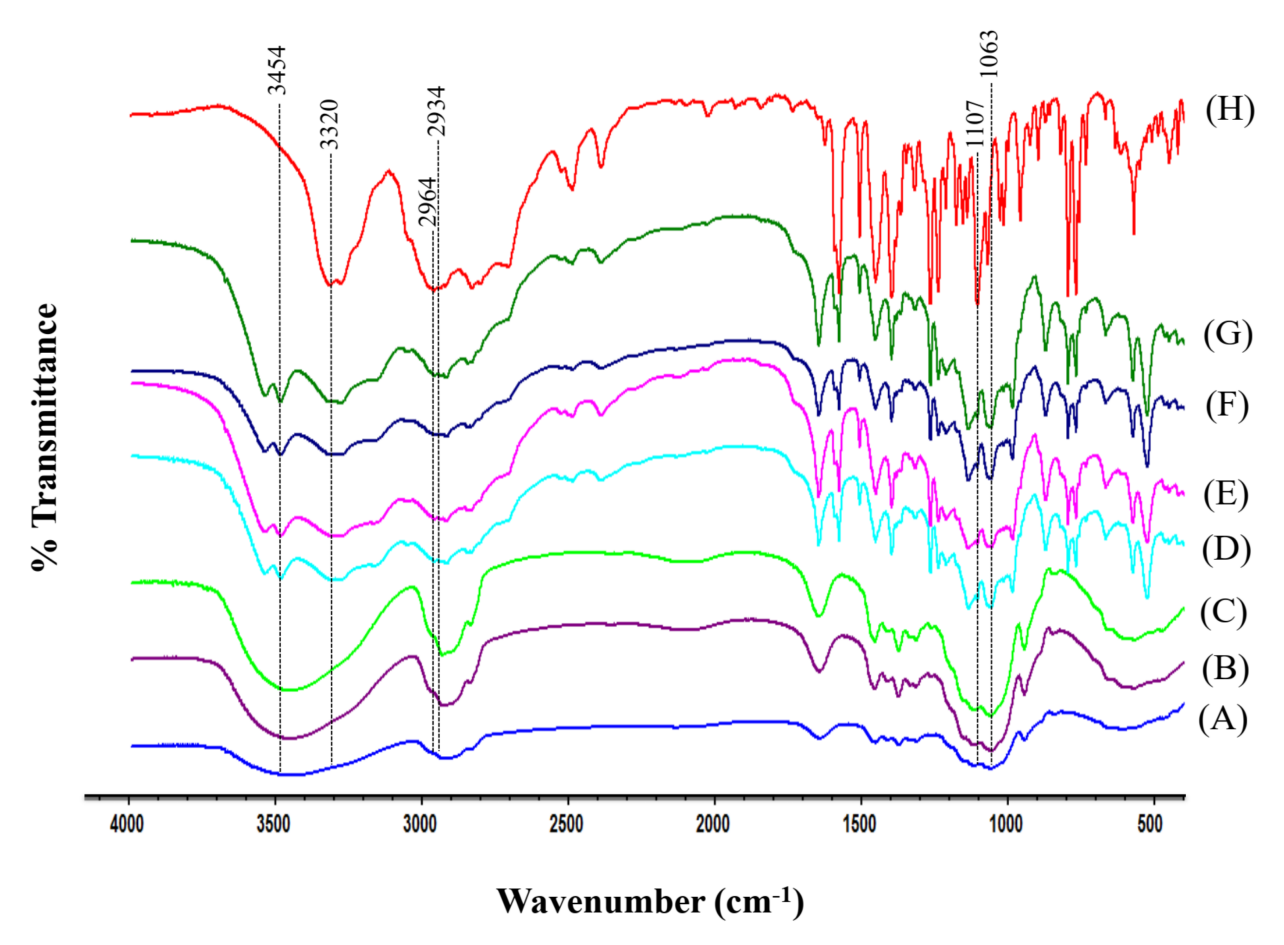
FT-IR spectra of H-K4M (**A**), H-K15M (**B**) H-K100M (**C**), Tab-K4M (**D**), Tab-K15M (**E**), Tab-K100M (**F**) Tab-Mixed (**G**), and PNL (**H**).

FT-IR spectroscopy was used to look for possible interactions between PNL and the excipients in tablet formulations. The FT-IR spectra shown in Figure 2 revealed the characteristic bands associated with the chemical structure of PNL and the excipients used in the compressed HPMC-based matrix tablets. It could be seen that the FT-IR spectra of distinct HPMC types were similar. Although H-K4M, H-K15M, and H-K100M are different viscosity grades, all these HPMC types have identical degrees of methoxyl and hydroxypropoxyl substitutions. Therefore, the FT-IR spectra of these distinct HPMC types did not show any significant difference. In the fingerprint region, the broad characteristic band around 1063 cm^−1^ represented the signals from C–O–C stretches, secondary alcohols, and methoxy groups in HPMC [28]. The FT-IR spectra of all HPMC types give the broad peak at 3454 cm^−1^ and the characteristic peak at 2934 cm^−1^ representing the presence of O–H stretching and the vibration of the C–H group, respectively [29]. The spectrum of PNL shows the characteristic band of the secondary hydroxyl group at 3320 cm^−1^, the vibration of the secondary amine group at 2964 cm^−1^, and the C–O–C stretching in aryl alkyl ether at 1107 cm^−1^ [30]. For all tablet formulations, the appearance of a doublet in the region 3400–3600 cm^−1^ can be assigned to the O–H stretching modes of the crystallization water in the dibasic calcium phosphate dihydrate molecule [31]. The wavenumbers of the functional groups of PNL remained intact in the tablets, indicating no prominent interaction between PNL and other excipients. 

**Figure 3:**
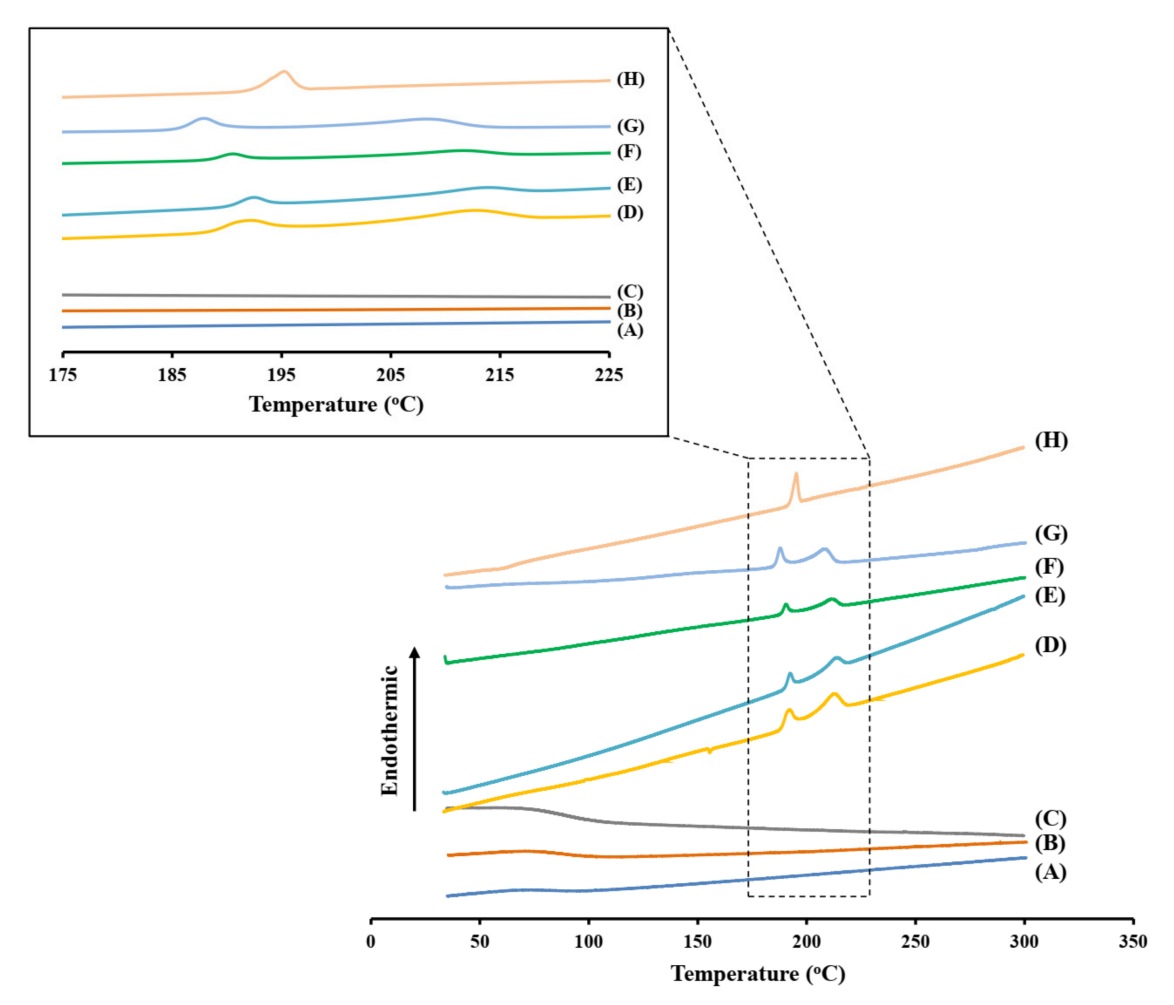
DSC thermograms of H-K4M (**A**), H-K15M (**B**) H-K100M (**C**), Tab-K4M (**D**), Tab-K15M (**E**), Tab-K100M (**F**) Tab-Mixed (**G**), and PNL (**H**).

A DSC investigation was conducted on the PNL sample, powder of various HPMC grades, and each tablet formulation. According to DSC thermograms (Figure 3), all used HPMC grades (H-K4M, H-K15M, and H-K100M) show no melting peak upon heating up to 300 °C. These DSC thermograms reflect the amorphous nature of HPMC, and the DSC results of these HPMC grades agree with what is reported in the literature [32]. The endothermic peak in the DSC scan of PNL in Figure 3H corresponds to the melting temperature of the pure enantiomer of PNL at 195.23 °C [33,34]. For Tab-K4M, Tab-K15M, Tab-K100M, and Tab-Mixed (Figure 3D–G), the broad endotherm was observed in the temperature range between 200 and 220 °C. It can be attributed to dehydration and water vaporization from dibasic calcium phosphate dihydrate [35,36]. The endothermic peak of PNL in all tablet formulations reflected the thermal characteristics of PNL alone. As a result, it was assumed that there was no evidence of interaction between PNL and the excipients.

**Figure 4:**
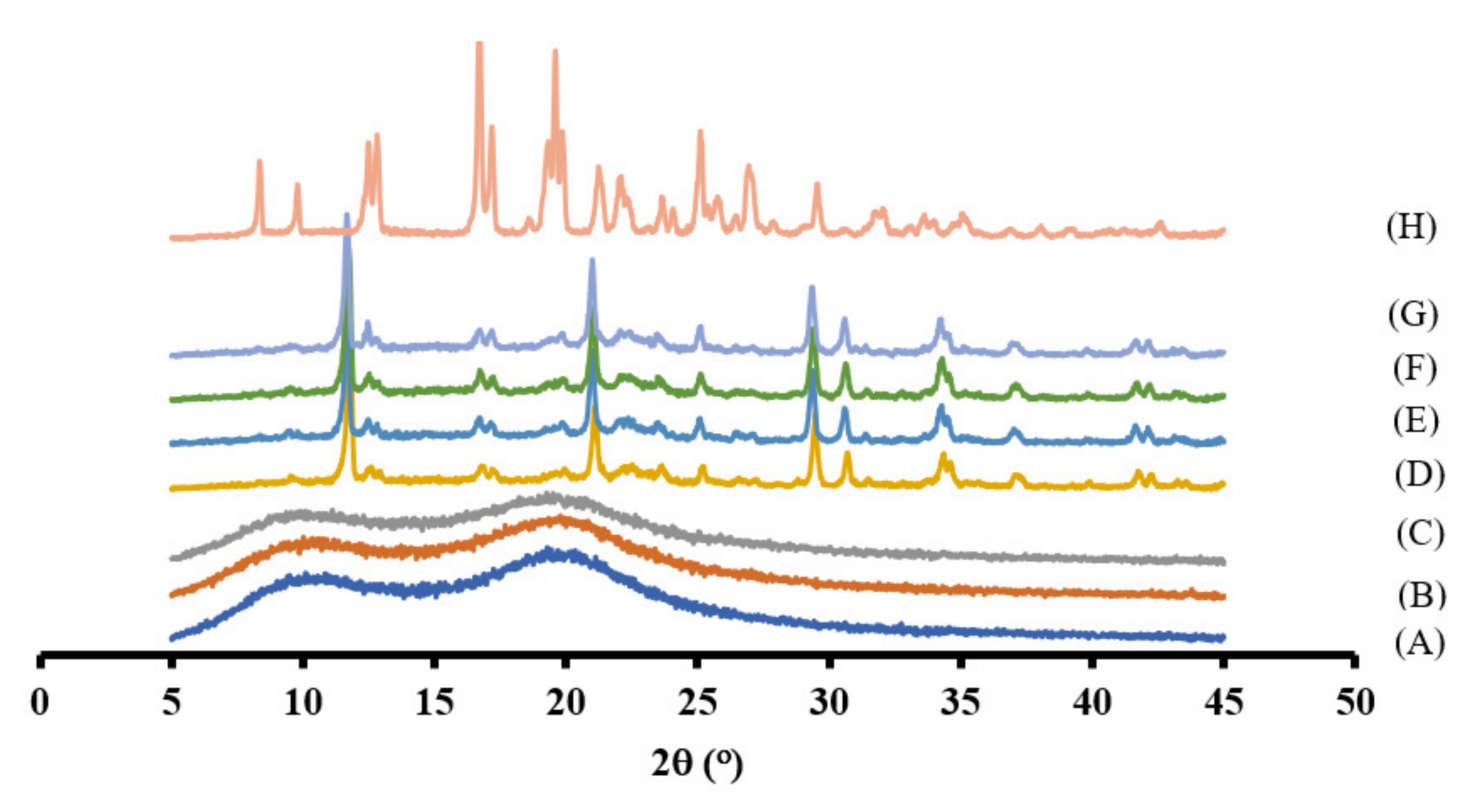
PXRD patterns of H-K4M (**A**), H-K15M (**B**) H-K100M (**C**), Tab-K4M (**D**), Tab-K15M (**E**), Tab-K100M (**F**) Tab-Mixed (**G**), and PNL (**H**).

PXRD results of pure PNL, powder of various HPMC grades, and each tablet formulation were given in Figure 4. PNL’s PXRD pattern revealed multiple high-intensity peaks, indicating the drug’s crystalline structure. The absence of a diffraction peak in PXRD patterns of various HPMC grades indicated that HPMC was amorphous. PNL had multiple high-intensity peaks in the PXRD patterns of all drug-containing tablets, meaning that PNL and the excipients were compatible. The PXRD results agreed with the FT-IR and DSC data, confirming that PNL and the used excipients were compatible.

### 3.2. SEM

**Figure 5:**
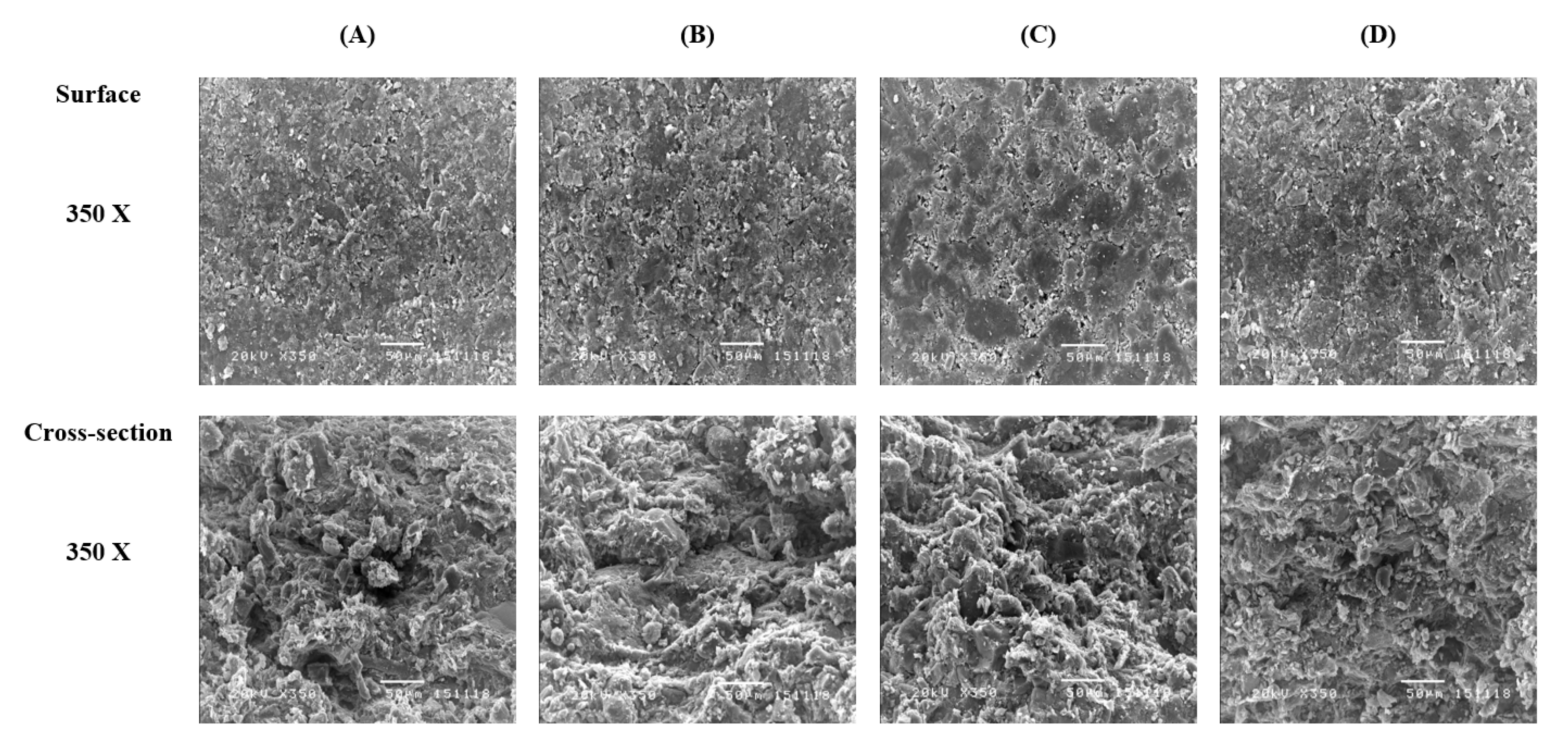
SEM images of Tab-K4M (**A**), Tab-K15M (**B**), Tab-K100M (**C**), and Tab-Mixed (**D**).

The scanning electron microscopy (SEM) imaging technique can provide data on the surface morphology of the tablet’s outer surface or cross-sections [37,38]. The tablets’ surface morphology was rough, with aggregated particles and small holes, and cracks were visible in cross-sections, as shown in Figure 5. Tab-K4M and Tab-K15M had dense, continuous masses with some irregularities, while Tab-K100M had the roughest breaking surface. The surface of Tab-Mixed appeared to be made up of a mix of a continuous dense mass and rough-breaking pieces. The images also emphasized that the inner structures of all tablets were composed of solid composition and air, known as tablet porosity [39]. The tablet porosity can be considered a bulk feature that impacts liquid and drug transport through the solid dosage form [38]. The compression force and tablet composition may affect the surface area of the pores and the number of distinct pore diameters in the tablets. However, the compression force used in this study was fixed. The effect of HPMC types on the porosity was further investigated using mercury porosimetry. 

### 3.3. Porosity

Mercury porosimetry is based on mercury penetration into the sample [40]. The mean pore diameter and pore size distribution significantly impact liquid penetration into porous material [41]. The pore range detectable by mercury porosimetry covers a wide pore diameter range. Mercury porosimetry can detect large intergranular pores as well as small intragranular and interparticle pores [40]. In addition, the pore size distribution of compressed tablets made from powders or granules can be determined using mercury porosimetry [26,40]. 

The pore size distribution curves can be represented in the following two formats: pore diameter vs. log differential intrusion for a broad pore distribution range and pore diameter vs. differential intrusion for capillary pore distribution [42].

The A_tot_ values of Tab-K4M, Tab-K15M, Tab-K100M, and Tab-Mixed were 5.840, 6.917, 1.136, and 1.156 m^2^/g, respectively. The number of pores with a minimal diameter significantly impacts the total pore area determined by mercury porosimetry [40]. It can be presumed that the compression of HPMC-K4M and HPMC-K15M formulations produces some small pores in the tablets. The D_m_ of Tab-K4M, Tab-K15M, Tab-K100M, and Tab-Mixed were 0.06521, 0.06433, 0.30179, and 0.32479 μm, respectively. The smaller pores substantially influence the mean pore size [40]. According to the IUPAC classification, mesopores and macropores have diameters of 2–50 nm and greater than 50 nm, respectively [18]. The mean pore diameter data revealed the presence of macropores in the matrices of all tablet formulations. The median pore diameter of Tab-K4M, Tab-K15M, Tab-K100M, and Tab-Mixed were 0.41738, 0.43225, 0.49200, and 0.52986 μm, respectively. Tab-K100M and Tab-Mixed had a larger median pore diameter than Tab-K4M and Tab-K15M. Because median pore diameter is based on pore volume, a decrease in median pore diameter means that the volume of small pores increased relative to large pores [27].

**Figure 6:**
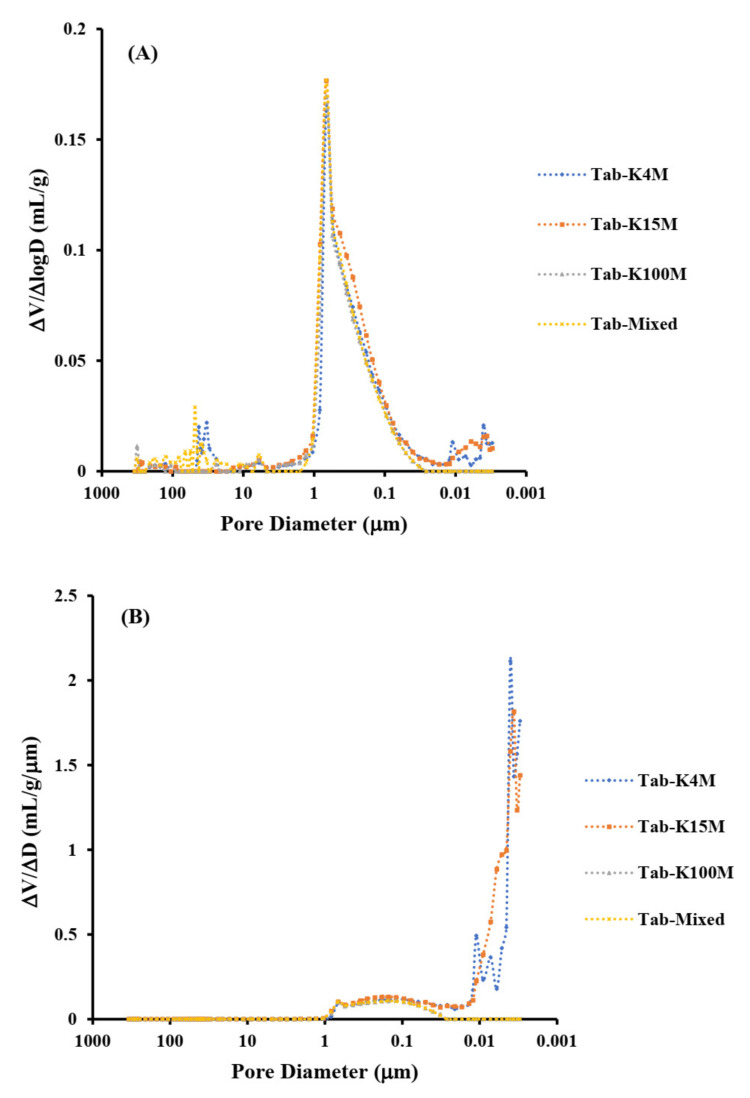
Logarithmic volume size distribution (ΔV/ΔlogD) of compressed HPMC-based matrix tablets (**A**) and pore volume size distribution (ΔV/ΔD) of compressed HPMC-based matrix tablets (**B**).

Figure 6A shows the log differential intrusion as a function of the pore diameter of the compressed HPMC-based matrix tablets. The logarithmic transformation (ΔV/ΔlogD) overemphasizes the large pore fraction [43]. Therefore, the size distribution in the large pore range can be compared using the logarithmic differential intrusion plotted against the pore diameter. According to the pore size distribution (Figure 6A), Tab-K4M, Tab-K15M, Tab-K100M, and Tab-Mixed all have a major peak in the pore range of 0.02-2 μm, and the peak values of the pore size distribution are quite close to each other. For Tab-K4M and Tab-K15M, some contributions from the small pore appeared at the tail of the pore size distribution curves at the small diameter side. The pore size distribution curves were shown as pore diameter vs. differential intrusion (ΔV/Δ D) to establish the pore size distribution in the smaller pore diameter region (Figure 6B). Only Tab-K4M and Tab-K15M possessed the small pores (diameter < 0.01 μm) observed in the pore size distribution curves (Figure 6B). The lower viscosity grade of HPMC in the HPMC K-series was thought to deform during compression, filling inter-particulate voids [44]. This phenomenon could be because low viscosity HPMC has shorter polymeric chains and easily deforms. This phenomenon may contribute to a small pore proportion in Tab-K4M and Tab-K15M.

### 3.4. Drug Release

**Figure 7:**
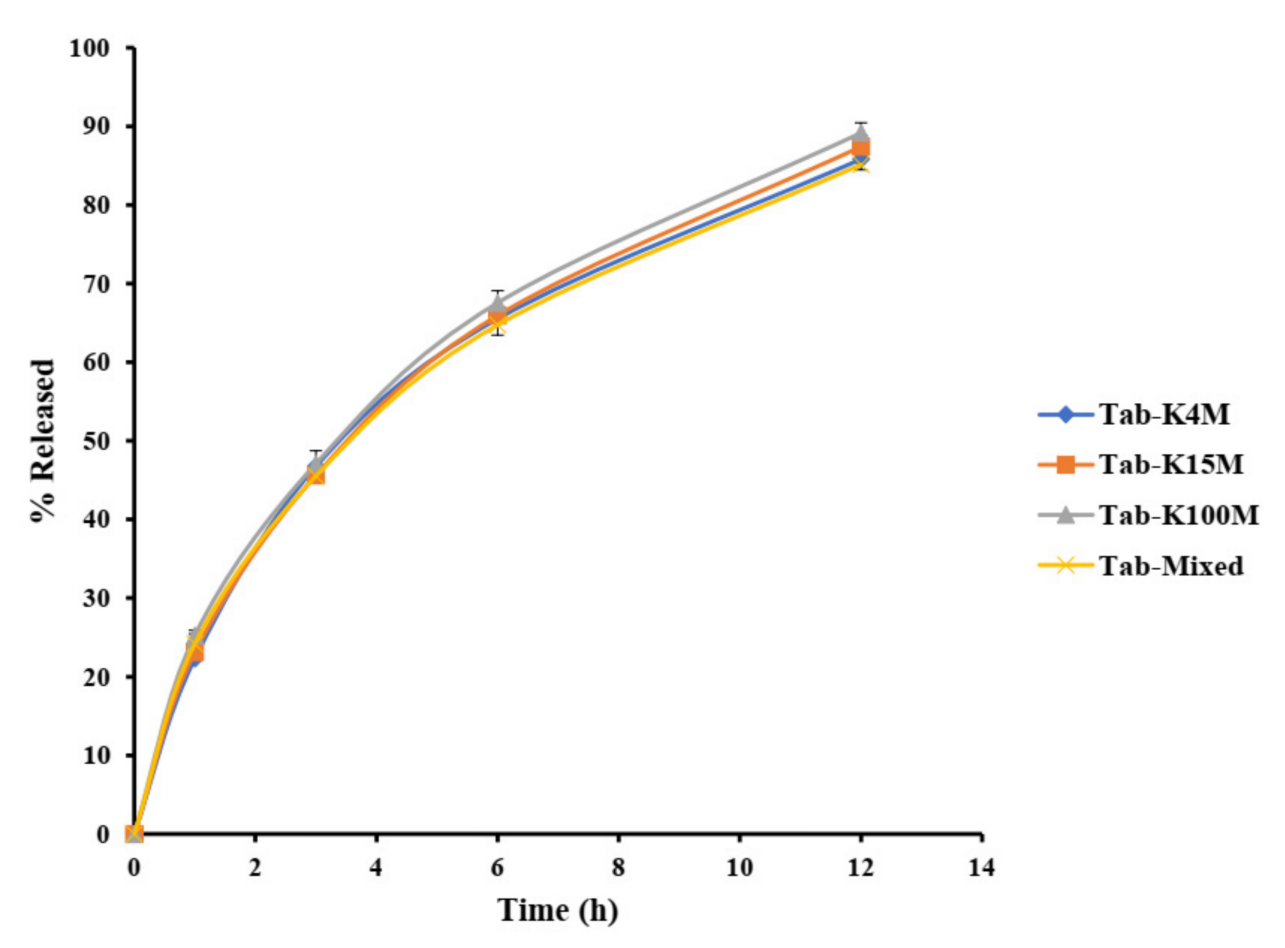
In vitro release of PNL from compressed HPMC-based matrix tablets.

The release profiles of PNL and the total amount of PNL released at each time point are shown in Figure 7 and Table 2, respectively. It was found that the drug release was sustained for more than 12 h in all matrices. Table 3 shows the Higuchi and Korsmeyer–Peppas kinetic models, as well as their fitting parameters, for drug release data. Both models were well-suited to all compressed HPMC-based matrix tablet formulations. Tab-K4M, Tab-K15M, Tab-K100M, and Tab-Mixed all had comparable Higuchi constants. These may imply that the viscosities of the hydrated matrices may be identical, although there are differences in their viscosity grades [18]. It has been reported that the release of the water-soluble drug was independent of the viscosity grade of polymer in the HPMC K-series [45]. However, the highest R^2^ value was observed for the Korsmeyer-Peppas model, indicating that the release data of all HPMC matrices were best fitted with the Korsmeyer–Peppas model. The Korsmeyer–Peppas model provides insight into the drug release mechanism in the swellable polymeric matrices [8]. Although all matrix tablet formulations fit the Higuchi equation well, the n values from the Korsmeyer–Peppas model for all matrices were higher than 0.45, representing the anomalous transport. These suggested that the drug release from these matrices was not diffusion controlled. According to the n values, the release of PNL from these compressed HPMC-based matrix tablets was governed by the combination of the drug diffusion through the gel layer and the erosion of the polymer gel layer. HPMC is a hydrophilic polysaccharide that can swell and is soluble in water [46]. The processes of diffusion and matrix erosion can occur simultaneously [18]. Therefore, the matrix erosion and drug diffusion phenomena are involved in drug release.

Table 4 shows the times required to release 25% (T_25_), 50% (T_50_), and 75% (T_75_) of PNL from various matrix tablet formulations. The differences in the release of PNL from the distinct compressed HPMC-based matrix tablet formulations were relatively small. However, according to release parameters T_50_ and T_75_, the release of PNL from Tab-K100M was significantly faster than the others (*p* < 0.05). The difference in T_25_ values of Tab-K100M and Tab-Mixed was not statistically significant (*p* > 0.05), while the T_25_ values of Tab-K4M and Tab-K15M were significantly higher than that of Tab-K100M (*p* < 0.05). Several research works reported that the pore size influenced the drug release, and the matrix with the greatest pore diameter had the fastest drug release [47]. When a drug molecule is exposed to the release media, it dissolves and diffuses through the media-filled pores, releasing the drug from a porous drug delivery matrix [48]. The drug release from the matrix was expected to be retarded by the small pore size [49]. However, the combined effect of the diffusion and erosion mechanisms was responsible for the PNL release from the matrix tablets. Water uptake and erosion may cause a change in drug diffusion path length in the release medium. Therefore, the PNL release from Tab-Mixed differed from Tab-K100M after the initial dissolution stage.

## 4. Conclusions

Compressed HPMC-based matrix tablets containing PNL were manufactured by direct compression. According to a compatibility study, HPMC and chosen excipients were compatible. Mercury porosimetry was used to determine the porosity of the tablets. The tablets made up of low viscosity HPMC contain tiny pores. Shorter polymeric chains are more likely to deform during compression, resulting in a small pore proportion. PNL was released continuously for more than 12 h. The differences in PNL release between the various formulations of compressed HPMC-based matrix tablets were relatively minor. Nevertheless, based on the release parameters T_50_ and T_75_, PNL release from Tab-K100M was significantly faster than that of the other formulations. An anomalous controlled-release mechanism governed the sustained PNL release from the compressed HPMC-based matrix tablets. As a result, the various types of HPMC could be beneficial for the extended release of PNL from compressed HPMC-based matrix tablets. The preparation of HPMC-based matrix tablets is a potential and economically favorable way of developing oral extended-release matrices in the future. 

## Figures and Tables

**Table 1:** Formulation of compressed HPMC-based matrix tablets.

Formulation	HPMC (mg)		
	H-K4M	H-K15M	H-K100M
Tab-K4M	60	0	0
Tab-K15M	0	60	0
Tab-K100M	0	0	60
Tab-Mixed	20	20	20

**Table 2:** The total amount of PNL released at each time point from compressed HPMC-based matrix tablets.

Formulation	Released (mg ± SD)			
	T_1_ = 1 h	T_2_ = 3 h	T_3_ = 6 h	T_4_ = 12 h
Tab-K4M	17.84 ± 0.94	37.40 ± 0.70	52.42 ± 1.29	68.66 ± 0.57
Tab-K15M	18.48 ± 0.49	36.49 ± 0.70	52.75 ± 0.78	69.93 ± 1.15
Tab-K100M	20.20 ± 0.53	37.67 ± 1.33	54.04 ± 1.24	71.33 ± 1.03
Tab-Mixed	19.33 ± 1.03	36.41 ± 0.48	51.78 ± 1.03	68.11 ± 0.53

**Table 3:** Higuchi and Korsmeyer–Peppas model fitting of release data.

	Higuchi Equation		Korsmeyer–Peppas Equation		
	k_H_ (min^−^^1^^/^^2^)	R^2^	n	k (min^−n^)	R^2^
Tab-K4M	3.292	0.9852	0.487	3.569	0.9857
Tab-K15M	3.324	0.9914	0.497	3.384	0.9915
Tab-K100M	3.410	0.9926	0.481	3.839	0.9938
Tab-Mixed	3.265	0.9912	0.476	3.772	0.9929

**Table 4:** T_25_, T_50_, and T_75_ for the release of PNL from compressed HPMC-based matrix tablets.

	T_25_ (min) ^a^	T_50_ (min) ^a^	T_75_ (min) ^a^
Tab-K4M	54.67 ± 4.37	226.69 ± 9.32	521.25 ± 11.00
Tab-K15M	55.94 ± 2.37	225.51 ± 6.92	509.82 ± 14.10
Tab-K100M	49.46 ± 3.57	209. 02 ± 9.63	485.82 ± 16.51
Tab-Mixed	53.05 ± 3.30	227.26 ± 6.59	532.53 ± 9.96

^a^ T_25_, T_50_, and T_75_ are the time for 25, 50, and 75% of PNL release, respectively.

## Data Availability

Not applicable.
